# Real-life use of Doravirine in treatment-experienced people living with HIV: A multicenter Italian study

**DOI:** 10.1097/MD.0000000000029855

**Published:** 2022-07-29

**Authors:** Maria Mazzitelli, Melania Degli Antoni, Francesco Castelli, Diego Ripamonti, Gianluca Zuglian, Giuseppe Lapadula, Massimiliano Fabbiani, Alice Ferraresi, Cristina Putaggio, Anna Maria Cattelan, Eugenia Quiros-Roldan

**Affiliations:** a Infectious and Tropical Diseases Unit, Padua University Hospital, Padua, Italy; b Department of Infectious and Tropical Diseases, ASST Spedali Civili General Hospital and University of Brescia, Brescia, Italy; c Infectious Diseases Unit, ASST Papa Giovanni XXIII, Bergamo, Italy; d University of Milan Bicocca, Monza, Italy; e Infectious and Tropical Diseases Unit, Azienda Ospedaliero-Universitaria Senese, Siena, Italy; f Division of Infectious Diseases, Cremona Hospital, Cremona, Italy.

**Keywords:** doravirine, dyslipidemia, HIV, PLWH, real-life, switch

## Abstract

Use of doravirine (DOR), a new nonnucleoside reverse-transcriptase inhibitors recently approved for HIV treatment, is still unclear in clinical practice and real-life data are scarce.

We retrospectively investigated the rationale for switching people with HIV to DOR-containing/-based regimens in a real-life cohort. Among 132 patients (68.9% males, median age 56 years), the main reasons to start DOR were prevention of toxicities (39.4%) and dyslipidemia (18.2%). DOR was combined with integrase inhibitors in 40.9% cases, and in 25.7% of patients, DOR was prescribed without availability of a genotypic resistance test.

Twenty-four weeks after the switch to DOR-containing/-based regimens, no significant changes in CD4+ T-cell count, CD4/CD8 ratio, detectable HIV-RNA, serum creatinine levels, and body weight were detected. By contrast, a significant reduction in lipids (both cholesterol and triglycerides) was observed in 52 patients for whom a follow-up assessment was available (*P* = .008 and .01, respectively).

Our data confirmed that switching to DOR-containing/-based regimens may have a favorable impact on lipid profile and a neutral impact on weight gain. However, more data are needed to support its use in patients who do not have a genotypic test available or have an extensive nonnucleoside reverse-transcriptase inhibitors-associated resistance, as well as its use in a dual regimen, especially in combination with second-generation integrase inhibitors.

## 1. Introduction

Doravirine (DOR) is a new-generation nonnucleoside reverse-transcriptase inhibitor (NNRTI) recently approved for the treatment of human immunodeficiency virus 1 (HIV 1).^[1]^ DOR, which is available both as a single-tablet (combined with tenofovir disoproxil fumarate and lamivudine) or as a single agent, is recommended in combination with other antiretroviral agents both in treatment-experienced and in naive people living with HIV (PLWH).^[2]^ Despite the availability of several highly effective and well-tolerated antiretroviral regimens, DOR may still be a valuable additional option in the treatment armamentarium, due to its safety profile, tolerability, minimal drug–drug interaction, and its activity against viruses harboring the major resistance mutations selected by the other available NNRTIs.^[3,4]^ Moreover, DOR provided good pharmacokinetics forgiveness over 72 hours after drug interruption in healthy volunteers.^[5]^ Data from the clinical trials showed also that DOR has little or no impact on lipids, and it could be of help for subjects with metabolic issues.^[6–8]^ In addition, in aging people comorbidities (especially the metabolic ones) and polypharmacy (possibly perpetrating drug interactions) represent 2 important challenges when selecting HIV treatment, which could benefit from the availability of drug such as DOR.^[9,10]^ Several antretroviral regimen options are possible, both in a naive setting and in switching strategies since many agents are available to clinicians (particularly when there are no resistance mutations in the background).^[2,11]^

However, when the genotype confirms the presence of resistance mutations, the choices get narrower and more complex regimens are needed. DOR has not been studied in the setting of resistance mutations; clinical trials have compared this new NNRTI with boosted darunavir (bDRV) and efavirenz (EFV) in naive setting,^[6,7,12]^ and have also explored its role in switching strategies.^[8,13]^

Although DOR efficacy on viruses harboring major NNRTI resistance mutations, such as K103N, Y181C, and G190A, has been shown in vitro, no clinical data are currently available regarding the use of DOR in patients infected with drug-resistant HIV. Thus, current guidelines do not recommend DOR use in this context.^[2]^ Nonetheless, clinical practice might diverge from this recommendation.

Data regarding the use of this new NNRTI in a real-life setting are still scarce,^[14–16]^ and it is important to define how clinicians have interpreted the role of DOR in the current HIV armamentarium. Therefore, this study aimed to describe the reasons leading clinicians to switch patients to a DOR-containing regimen and its role in clinical practice. Second, we aimed at assessing the changes in virological, immunological, and metabolic parameters from baseline to week 24 after the switch.

## 2. Methods

This is a retrospective multicentre cohort study, including 6 Italian HIV centers (Bergamo, Brescia, Cremona, Monza, Padua, and Siena).

In Italy, the use of DOR was approved in January 2020. All PLWH (older than 18 years), who switched to DOR-containing regimens from February 1, 2020, to December 31, 2021, were included. The study was conducted accordingly to Declaration of Helsinki and to principles of good clinical practice. It included retrospectively collected and anonymized data. Retrospective studies including such kind of data, as per Italian law (Italian Drug Agency note, March 20, 2008, no. 76), are waived both from patient’s consenting and Ethical Approval.^[17]^ Moreover, for this study, we used the general authorization of the Italian Guarantor for the use of anonymized demographical and clinical data. For each patient, we collected demographics (age, gender, ethnicity), clinical (length of HIV disease, body weight, antiretroviral therapy, intake of lipid-lowering agents), and laboratory data (HIV-RNA viral load measured by Cobas Real Time Polymerase Chain Reaction by Roche, CD4+T-cell count—measured by cytofluorimetry, creatinine, cholesterol, and triglycerides levels). Furthermore, we gathered all data available regarding HIV major resistance mutations for each antiretroviral class as per Stanford University HIV Drug Resistance Database.^[18]^ Resistance test available before patients started any antiviral agent was performed by using the Sanger sequencing methods for sequencing of plasma HIV-RNA. As per EACS guidelines 2021, “confirmed virological suppression” was defined as a double consecutive detection of HIV viral load <50 copies/mL within the previous 6 months.^[2]^ Reasons for DOR introduction were obtained from patient health records and categorized as follows: proactive switch (i.e., toxicity prevention in patients not experiencing any overt side effect with the current regimen), dyslipidemia, weight gain, other ongoing toxicity or undesired side effects with current antiretrovirals, virological failure, and management of drug-to-drug interactions. Among patients with at least 6 months of follow-up after DOR introduction, we compared lipids, body weight, creatinine, and viroimmunological parameters (viral load, CD4+ T-cell count, and CD4/CD8 ratio), before switch and 24 weeks after.

Continuous variables were compared by Student t test for normally distributed variables and the Mann–Whitney U test for nonnormally distributed variables. Categorical variables were evaluated using the Chi-squared or 2-tailed Fisher exact test. Values for continuous variables were expressed as median [interquartile range IQR)] or as percentages for categorical variables. Two-tailed tests were used to determine statistical significance; a *P* value of <.05 was set as the level of significance. Statistical analysis was performed using STATA software, version 11 (Stata Corporation, College Station, TX).

## 3. Results

Over the study period, 132 PLWH were switched to DOR-containing regimens (Table 1 shows the main baseline characteristics). A total of 91 of 132 (68.9%) of patients were male and median age was 56 years (IQR: 51–61). Median duration of HIV infection was 22 years (IQR: 12–30), while median baseline CD4+ T-cell count was 654 (IQR: 400–890) cells/mm^3^. At the time of switch, 107 patients (81.1%) had an undetectable HIV viral load, while 18.9% of subjects had plasma HIV-RNA >200 copies/mL (IQR: 664–7280). At baseline, according to historical cumulative genotypes, nucleoside reverse-transcriptase inhibitor (NRTI) and NNRTI resistance mutation rates were 21.9% and 21.2%, respectively, while resistance rates for integrase inhibitor (INI) and protease inhibitor (PI) were, respectively, 3.8% and 4.5%. Among 28 subjects who had NNRTI resistance mutations, 17 (60.7%) had K103N and 3 (10.7%) had Y181C. Six patients (21.4%) had the E138A accessory mutation, selected in patients who received rilpivirine or etravirine.

**Table 1:** Main clinical and biochemical parameters at baseline.

Characteristics	Overall, n = 132, n (%)
Gender, male	91 (68.9)
Age, y, median (IQR)	56 (51–61)
Years since HIV diagnosis, median, (IQR)	22 (12–30)
CD4+ T-cell count, cells/mm^3^, median (IQR)	654 (400–890)
CD4/CD8 ratio*	0.75 (0.5–1.1)
HIV-RNA	
<50 copies/mL	107 (81.1)
50–200 copies/mL	9 (6.8)
>200 copies/mL	16 (12.1)
Resistance test	
Genotype not available	34 (25.7)
Genotypic major mutations	
INI	5 (3.8)
PI	6 (4.5)
NRTI	29 (21.9)
NNRTI	28 (21.2)
No mutations	37 (28)
In 2 antiretroviral classes	20 (15.1)
In 3 antiretroviral classes	11 (8.3)
In 4 antiretroviral classes	2 (2.3)
Antiretroviral regimen before switch	
2NRTI + INI	30 (22.7)
2NRTI + NNRTI	19 (14.4)
2NRTI + PI	18 (13.6)
NNRTI + INI	3 (2.3)
NRTI + INI	10 (7.6)
INI + PI	27 (10.5)
NRTI + PI	4 (3)
Other	21 (15.9)
Reason for switching	
Drug–drug interaction	17 (12.9)
Ongoing toxicity	9 (6.8)
Dyslipidemia	24 (18.2)
Weight gain	7 (5.3)
Proactive switch	52 (39.4)
Virological failure	23 (17.4)
Agents associated to DOR at switching	
INI	54 (40.9)
Dolutegravir	45 (34)
Raltegravir	9 (6.9)
PI	4 (3)
Atazanavir/cobicistat	1 (0.7)
Darunavir/ritonavir	3 (2.3)
NRTI	57 (43.2)
Abacavir/lamivudine	3 (2.3)
Tenofovir alafenamide/emitricitabine	25 (18.9)
Tenofovir disoproxil fumarate/emtricitabine	4 (3)
Tenofovir disoproxil fumarate/lamivudine	25 (18.9)
PI+INI (boosted darunavir+dolutegravir)	8 (6.1)
Other regimes	9 (6.8)

Overall, the prevalence of subjects with resistance at 2 or more classes (described as “heavily treatment-experienced patients” according to recent definitions) was 31%.^[19]^ No patients had major mutations for DOR at baseline. However, a 50-year-old highly treated male (for whom DOR was combined with lenacapavir, dolutegravir, bDRV, and tenofovir alafenamide), showed the V108X mutation which causes a low-level reduction in susceptibility to DOR.^[20]^

The main reasons for switching to DOR-containing regimens were proactive switch (39.4%), dyslipidemia (18.2%), virological failure (17.4%), and managing drug-to-drug interactions (12.9%). Seven patients (5.2%) were switched to DOR-containing regimens for significant weight gain (they were previously on treatment with INI in 2 of 7 cases, with boosted PI in 2 of 7 cases, and with 2NNRTI+NRTI in 3 cases). Among patients switched from a PI-based regimen to a DOR-containing regimens, 14 of 24 (58.3%) and 13 of 17 (76.5%) were switched off from bDRV for dyslipidemia and ongoing drug-to-drug interactions perpetrated by the presence of the boosting agents, respectively.

Nine patients (6.8%) were switched for ongoing toxicity. In particular, in 7 of 9 cases toxicity was associated with an INI-based regimen (gastrointestinal symptoms and central nervous system side effects such as headache and insomnia).

DOR was often administered with 2NRTIs (43.2% cases) or as a dual therapy along with INIs (in 40.9% cases), mostly dolutegravir (45/54 cases). In 6.1% cases, it was associated with both a PI and an INI, while in 6.8% it was combined with multiple different drugs.

Treatment with DOR was discontinued during the first month in 5.3% (7/132) patients for the following reasons: 2 virological rebounds due to scarce adherence, 2 due to gastrointestinal intolerance, 1 due to central nervous system side effects (DOR in this latter case was associated with dolutegravir, and the patient was previously on boosted atazanavir and lamivudine), 1 because of difficulties in swallowing tablets, and 1 due to headache.

Fifty-two patients were followed for at least 24 weeks after DOR introduction. Differences in parameters after baseline are summarized in Table 2.

**Table 2:** Change from baseline to week 24 of main clinical characteristics for 52 patients who reached the follow-up point.

Parameter	Baseline, n (%)	Week 24 after switch n (%)	*P* value
Detectable HIV-RNA (Yes)	4/52 (7.6)	3/52 (5.7)	.695
CD4+, cell/mm^3^, median (IQR)*	612 (423–783)	625 (440–835)	.708
CD4/CD8 ratio	0.73 (0.5–1.2)	0.72 (0.5–1.2)	.160
Cholesterol, mg/dL, median (IQR)	205 (171–232)	193 (155–214)	.008
Triglycerides, mmol/L, median (IQR)	116 (89–172)	114 (82–156)	.01
Body weight, kg, median (IQR)*	78.5 (67–84)	76.5 (67–83)	.093
Creatinine, mg/dL, median (IQR)	0.99 (0.8–1.2)	1 (0.8–1.2)	.323

No differences were observed regarding the proportion of subjects with virological suppression, CD4+ T-cell count or CD4/CD8 ratio, detectable HIV-RNA, and serum creatinine levels. A significant decrease was found in both cholesterol and triglycerides levels (*P* values = .008 and .01, respectively). A nonsignificant body weight reduction was observed in the subgroup of 38 patients who measured weight before and 24 weeks after the switch [on average, 78.5 (67–84); 76.5 (67–83) kg; *P* = .093].

## 4. Discussion

To the best of our knowledge, this is one of the largest real-life studies on the use of DOR. The main drivers for switching to DOR-containing regimens in our cohort were the intent to prevent metabolic complications (proactive switch) and dyslipidemia, followed by virological issues. This approach is consistent with other studies, in whom the main reasons for the switch were high cardiovascular/metabolic risk, treatment simplification, drug-to-drug interaction and toxicity.^[14–16]^ This suggests that clinicians look at DOR as a drug useful to improve the management of metabolic issues in clinical practice.

In our study, the prevalence of patients with multiple historical genotypic resistances was high, but DOR was selected only when no major mutations compromising its efficacy were present.^[18,21]^

While the most recent international guidelines suggest using DOR in combination with 2 NNRTIs as a first line antiretroviral regimen^[2,11]^ with a baseline resistance testing, in our experience DOR was used only for switching strategies. In 25.7% of patients, DOR was prescribed without a genotypic resistance test, but with a long history of persistently undetectable HIV-RNA.

Moreover, in our study a large proportion of patients (40.9%) was switched to DOR+INI, despite the absence of data exploring this 2-drug combination in clinical trials.

This may suggest that clinicians have a certain level of confidence in exploiting the high genetic barrier of DOR (which is higher than other NNRTIs) and its innovative resistance profile when there is no available cumulative genotype or as a rescue therapy in patients with limited options and complex clinical history, although clinical trials provided data limited only to naive patients or switched patients with previously undetectable HIV-RNA. Moreover, according to previously published data, we know that DOR resistance is uncommon in PLWH who have been treated with NNRTI.^[22]^ Indeed, data from ARCA cohort showed how on samples coming from 6893 patients who were NNRTI experienced prevalence of high or intermediate level of resistance to NNRTIs was of 6.1% and 12.7%, respectively.^[22]^ However, these authors showed how the previous exposure to efavirenz and etravirine was significantly associated with a high probability of detecting a DOR high-level resistance, while rilpivirine was associated with a very low probability. Due to the very low prevalence of DOR resistance, results from this study support the use of DOR even in NNRTI experienced patients, but a note of caution should be paid in patients who were previously exposed to etravirine or efavirenz.^[22]^

Although follow-up is available only in a subgroup of patients and limited to 24 weeks, our data suggest a significant favorable impact on the lipid profile, confirming the results from both clinical trials and real-life settings, which compared DOR-based regimens versus boosted protease inhibitor-based regimens.^[6–8]^ Of course, when the treatment switch is toward a boosted PI-including regimen plus DOR, this possible benefit may be lost.

No statistically significant impact on body weight was detected after 24-week follow-up. In naive patients, clinical trials with DOR showed that weight changes were similar across all treatment groups (DOR, bDRV, and efavirenz) at week 96 [2.4 (1.5), 1.8 (0.7), and 1.6 (1.0) kg, respectively].^[23]^

Also in switch strategies, weight gain was not significant at 144-week follow-up.^[8]^ Whether switching to a DOR-based regimen in real-life may affect body weight or body composition, particularly among those who experience weight gain during treatment with other regimens, deserves to be further investigated.

Last, since PLWH are aging with an increasing number of comorbidities and polypharmacy, it remains to be established which is the best companion for DOR among the elderly, given that tenofovir disoproxil fumarate may affect renal function and bone metabolism.

In conclusion, DOR-based regimens may become more appealing because this strategy may be a good alternative for patients suffering from hypertriglyceridemia, hypercholesterolemia, and weight gain. Of note, more data are needed on the use of DOR in patients that do not have a genotypic test available (despite past virological failures) or have an extensive NNRTI-associated resistance.

As intriguing as the option of DOR combined with second-generation INI may be, no trials have yet explored this regimen. Therefore, as robust data are scanty, caution is advised.

## Author contributions

E.Q.-R. was involved in conceptualization. M.M. and M.D.A. were involved in original draft preparation and methodology data curation. MM was involved in formal Analysis. D.R., G.Z., G.L., A.F., C.P., F.C., M.F., A.M.C., and E.Q.-R. were involved in writing—review and editing. All authors have read and agreed to the published version of the manuscript.

## Footnotes

* (Available only for 121 patients).

* Available for 38 patients.
